# Clinical manifestations of resistance to thyroid hormone beta (RTHβ) syndrome in children – multicenter case series

**DOI:** 10.3389/fendo.2026.1972541

**Published:** 2026-10-15

**Authors:** Anna Fedorczak, Martyna Sokół, Iwona Beń-Skowronek, Magdalena Mierzwa, Agnieszka Zachurzok, Agnieszka Bogusz-Wójcik, Julia Przybylak-Ortega, Elżbieta Moszczyńska, Paulina Berezowiec, Artur Bossowski, Natalia Dąbek, Artur Mazur, Kamil Dyrka, Marek Niedziela, Magdalena Makowiecka-Wołek, Robert Śmigiel, Agnieszka Ulenberg, Małgorzata Myśliwiec, Renata Stawerska

**Affiliations:** 1 Department of Endocrinology and Metabolic Diseases, Polish Mother’s Memorial Hospital—Research Institute, Lodz, Poland; 2 Department of Pediatric Endocrinology and Diabetology with the Endocrinology and Metabolic Laboratory, Medical University of Lublin, Lublin, Poland; 3 Department of Pediatrics, Faculty of Medical Sciences in Zabrze, Medical University of Silesia, Zabrze, Poland; 4 Department of Endocrinology and Diabetology, Children’s Memorial Health Institute, Warsaw, Poland; 5 Department of Pediatrics, Endocrinology, Diabetology with a Cardiology Division, Medical University of Bialystok, Bialystok, Poland; 6 Institute of Medical Sciences, Medical College of Rzeszow University, University of Rzeszow, Rzeszow, Poland; 7 Doctoral School, Poznan University of Medical Sciences, Poznan, Poland; 8 Department of Pediatric Endocrinology and Rheumatology, Institute of Pediatrics, Poznan University of Medical Sciences, Poznan, Poland; 9 Department of Pediatrics, Endocrinology, Diabetology and Metabolic Diseases, Medical University of Wroclaw, Wroclaw, Poland; 10 Department of Pediatrics, Diabetology and Endocrinology, University Clinical Center in Gdansk, Gdansk, Poland; 11 Department of Pediatric and Adult Endocrinology, Medical University of Lodz, Lodz, Poland

**Keywords:** child, neurodevelopmental disorders, resistance to thyroid hormone beta, RTH, THRB gene, THRB gene mutation

## Abstract

**Introduction:**

Resistance to thyroid hormone (TH) β (RTHβ) is a rare genetic disorder caused by mutations in the TH receptor β gene (*THRB*) and characterized by elevated TH levels with non-suppressed thyroid-stimulating hormone (TSH) and variable clinical presentation. The aim of the study was to characterize the clinical and biochemical features of children with RTHβ, correlate them with genetic findings and therapeutic management, to raise clinicians’ awareness of the specific characteristics of the condition.

**Material and methods:**

This retrospective multicenter case series included 15 children with genetically confirmed *THRB* variants. Clinical, laboratory, imaging, and treatment data were analyzed retrospectively.

**Results:**

All the patients demonstrated elevated free thyroxine and free triiodothyronine concentrations with detectable, non-suppressed TSH serum level. Common findings included tachycardia (73%), neurodevelopmental disturbances (60%), and a lean phenotype (BMI <10th percentile, 53%). Misdiagnosis of primary hyperthyroidism occurred in 20% of cases. Most patients did not require pharmacological treatment or were managed with beta-blockers alone for symptomatic tachycardia control.

**Discussion:**

Pediatric RTHβ shows consistent biochemical features but marked clinical heterogeneity. Awareness is essential to ensure accurate diagnosis and avoid unnecessary therapy.

## 1 Introduction

Resistance to thyroid hormone (TH) beta (RTHβ) is a rare syndrome characterised by reduced tissue responsiveness to thyroid hormones, primarily due to heterozygous mutations in the TH receptor β (THRB) gene (1). This defect impairs the negative feedback loop in the hypothalamic-pituitary-thyroid axis, resulting in elevated free thyroxine (FT4) and triiodothyronine (FT3) levels alongside non-suppressed thyroid-stimulating hormone (TSH) serum concentration (2). Thyroid hormone receptors exist in two main isoforms, THRα and THRβ, which show tissue-specific distribution. THRα is predominantly expressed in the central nervous system, myocardium, skeletal muscle, and adipose tissue, whereas THRβ is mainly expressed in the liver, hypothalamus, and pituitary gland. This differential expression underlies the characteristic phenotype of RTHβ, in which tissues expressing THRβ exhibit relative resistance, while THRα-dominant tissues remain sensitive to circulating thyroid hormones.

Although many individuals remain clinically euthyroid, RTHβ exhibits marked phenotypic heterogeneity. Clinical manifestations may include tachycardia, goitre, behavioral disturbances and growth abnormalities in children (3). In pediatric patients, the presentation may be particularly variable, often overlapping with attention-deficit or other neurodevelopmental disorders (4, 5). The biochemical profile may lead to diagnostic uncertainty and misclassification as primary hyperthyroidism or, less commonly, TSH-secreting pituitary adenoma (4). Published pediatric series remain small, and data on the clinical course and management of children with RTHβ are limited (5).

The aim of the study was to characterize the clinical and biochemical features of 15 children with this rare disorder, correlate them with genetic findings and therapeutic management, to raise clinicians’ awareness of the specific characteristics of the condition.

## 2 Materials and methods

### 2.1 Study design and setting

This was a retrospective, multicenter case series study evaluating the clinical presentation, diagnostic work-up, and management of pediatric patients diagnosed with RTHβ. Patients were identified from pediatric endocrinology centers across Poland through a collaborative initiative of the Young Members Club of the Polish Society for Pediatric Endocrinology and Diabetology. Over an approximately two-year period, clinicians at pediatric endocrinology centers consulted colleagues and reviewed available records to identify all eligible pediatric patients known to their centers. No formal patient registry was used for case ascertainment; the identified cases were collated in a collaborative spreadsheet.

### 2.2 Study population and data collection

Eligible participants were children and adolescents aged 0–18 years with a genetically confirmed diagnosis of RTHβ. Clinical, laboratory, genetic, and imaging data and medical history and family medical history were collected retrospectively from medical records. Height and BMI percentiles and z scores were calculated using WHO Child Growth Standards for children aged <5 years, Polish OLA reference charts for children aged 5 to <7 years, and Polish OLAF reference charts for those aged 7–18 years (6, 7). Short and tall stature were defined as height SDS <−2 and >+2, respectively. Tachycardia was defined as a resting HR above the age-specific 95th percentile. Thyroid function tests were interpreted using age- and local laboratory-specific reference ranges provided by laboratories performing the tests. The assessment of neurological disorders was based on the available results of neurological and/or psychological/psychiatric consultations.

### 2.3 Genetic analysis

Genetic confirmation of RTHβ was established by sequencing of the *THRB* gene. Methods varied by center and included Sanger sequencing as well as targeted next-generation sequencing panels. Variants were classified according to the American College of Medical Genetics and Genomics/Association for Molecular Pathology (ACMG/AMP) 2015 guidelines and described using Human Genome Variation Society (HGVS) nomenclature (8, 9). Variant interpretation was supported by data from ClinVar, HGMD and relevant literature. All variants were annotated according to the MANE Select transcript for *THRB* (NM_001354712.2).

### 2.4 Statistical analysis

Categorical variables were summarized as counts and percentages. Continuous variables were summarized as medians with interquartile ranges or ranges, as appropriate. No inferential statistical analyses were performed.

### 2.4 Institutional review board statement

The requirement of ethical approval was waived by Bioethics Committee of the Polish Mother’s Memorial Hospital–Research Institute in Lodz for the studies involving humans because this retrospective study involved the analysis of anonymized data collected during routine clinical care and included no additional interventions or procedures (Opinion No. KB-45/2026). The studies were conducted in accordance with the local legislation and institutional requirements. The ethics committee/institutional review board also waived the requirement of written informed consent for participation from the participants or the participants’ legal guardians/next of kin because the study used fully anonymized retrospective data and no individual participant could be identified.

## 3 Results

### 3.1 Study group

Fifteen patients with genetically confirmed resistance to thyroid hormone β (RTHβ) from the nine centers across Poland were included in this multicenter analysis. The cohort consisted of 7 males (47%) and 8 females (53%). The syndrome was diagnosed between 1 and 12.5 years of age with the median age at diagnosis was 7.42 (1.75–10.75) years. The patients’ characteristic frequency of clinical manifestations is presented in **Table 1**. Anthropometric data in **Table 1** were obtained at the time of RTHβ evaluation.

**Table 1:** Clinical Manifestations of RTHβ.

Patient no.	Gender	Age at diagnosis (years)	Height centile	Height SDS	BMI centile	BMI z-score	Tachycardia	Goiter	Neurodevelopmental disorder
1.	M	1.00	38	−0,31	9	−1.35	Yes	No	Attention deficit-hyperactivity disorder Psychomotor hyperactivity
2.	F	1.25	70	0.54	71	0.55	No	Yes	Psychomotor hyperactivity
3.	M	1.42	22	−0.77	36	−0.36	Yes	No	No
4.	F	1.75	99	2.39	5	−1.67	No	No	Autism spectrum disorder, psychomotor delay
5.	F	2.58	80	0.85	<3	−2.67	Yes	Yes	Psychomotor hyperactivity Autism spectrum disorder, Psychomotor delay
6.	M	3.92	52	0.06	<3	−2.18	Yes	No	Psychomotor delay, Speech delay
7.	M	7.42	54	0.09	<3	−2.86	Yes	No	Psychomotor hyperactivity Autism spectrum disorder
8.	M	6.83	36	−0.36	<3	−2.33	Yes	No	Psychomotor hyperactivity Autism spectrum disorder
9.	F	9.00	14	−1.07	30	−0.56	Yes	No	No
10.	F	9.17	82	0.93	99	2.31	Yes	No	Psychomotor delay
11.	M	9.42	78	0.77	6	−1.52	Yes	Yes	No
12.	F	10.75	19	−0.89	66	0.27	No	Yes	No
13.	F	10.92	61	0.27	29	−0.56	Yes	Yes	No
14.	F	10.83	96	1.74	<3	−2.38	Yes	Yes	Anxiety, emotional lability
15.	M	12.50	65	0.38	96	1.71	No	Yes	No
Total 15	M=7 (47%); F=8 (53%)	Median 7.42 years (Q1: 1.75; Q3:10.75)	Short stature 0, Tall stature 1 (7%)		<3rd 5 (33%) >3d and <10^th^ 3 (20%), Total 8 (53%)		11 (73%)	7 (47%)	Total 9 (60%) Psychomotor hyperactivity 5 (33%) Psychomotor delay 4 (27%) Autism spectrum disorder 4(27%) ADHD 1 (7%)

Tachycardia was observed in 11 out of 15 patients (73%) whereas goiter was present in 7 patients (47%). Neurodevelopmental or behavioural findings were observed in 60% of our cohort, including psychomotor hyperactivity in five patients (33%), one of whom had a documented ADHD diagnosis; psychomotor delay and autism spectrum disorder were each documented in four patients (27%). Abnormalities in body mass were frequently observed. Five patients (33%) had a BMI below the 3rd percentile for age and sex, and three patients (20%) had BMI values ≥3^rd^, but below the 10th percentile (together 8 patients (53%) had BMI below 10 percentile), indicating a predominance of a lean phenotype. Tall stature was identified in one patient (7%), whereas no patients met the criteria for short stature.

Eleven patients (73%) presented with significant coexisting disorders or clinically relevant additional findings. These included heart defects (hypoplastic left heart syndrome, HLHS, n=1, and mitral valve regurgitation, n=2), epilepsy (n=1), type 1 diabetes mellitus (T1D; n=1), selective IgA deficiency (n=1), congenital cystic adenomatoid malformation of the lung (CCAM type 1; n=1), and premature closure of the anterior fontanelle (n=1) and growth hormone deficiency (n=1). In this patient, GHD had been diagnosed earlier based on peak GH concentrations below 10 ng/mL in two stimulation tests, and recombinant human GH treatment was ongoing at the time of RTHβ diagnosis. Moreover, one patient had myopia, tall stature, an arm span-to-height ratio of 1.02, pectus excavatum, and scoliosis. Another patient presented with hip and patellar dysplasia, genu valgum, scoliosis, malocclusion, and enamel hypoplasia, and is currently undergoing further genetic evaluation for a suspected hereditary connective tissue disorder.

### 3.2 Indications for laboratory evaluation

The reasons for initial laboratory evaluation varied across the cohort (**Table 2**). Thyroid function tests were most commonly performed due to symptoms suggestive of hyperthyroidism, which were reported in six patients (40%). A family history of diagnosed or suspected RTHβ prompted biochemical testing in five patients (33%). Preoperative qualification and psychomotor developmental delay were each reported as indications in two patients (13%). Less frequent indications included suspected short stature and premature fontanelle closure, each recorded in one patient (7%).

**Table 2:** Reasons for initial thyroid function testing in the study cohort.

Reason for testing	Patients, n (%)
Symptoms suggestive of hyperthyroidism	6 (40%)
Family history of diagnosed or suspected RTHβ	5 (33%)
Preoperative assessment	2 (13%)
Psychomotor developmental delay	2 (13%)
Suspected short stature	1 (7%)
Premature closure of the anterior fontanelle	1 (7%)

Percentages are based on all 15 patients. Some patients had more than one reason for testing.

### 3.3 Family history

A positive family history of diagnosed or suspected RTHβ was finally identified in more than half of the cases (8, 53%). The same *THRB* variant was confirmed in relatives of 4 patients (27%), including siblings, fathers and grandmother. In other 4 cases (27%), one of the parents presented with biochemical features consistent with RTHβ, although genetic confirmation had not been performed or was still under evaluation.

### 3.4 Laboratory findings at diagnosis

All patients with available biochemical data demonstrated findings consistent with resistance to thyroid hormone β (**Table 3**). FT4 and FT3 concentrations exceeded the upper limit of age- and local laboratory-specific reference ranges in all patients (median FT4 2.94 ng/dL; IQR 2.40–3.40; median FT3 9.10 pg/mL; IQR 7.71–11.27). TSH levels remained detectable and non-suppressed (2.72 mIU/L; IQR 2.20–3.89), with mildly elevated TSH concentrations observed in one patient. Anti-TSHR, anti-TPO and anti-thyroglobulin antibodies were negative in all patients.

**Table 3:** Thyroid function test results at diagnosis.

Patient no.	Age at diagnosis (years)	TSH (mIU/L)	FT3 (pg/mL)	FT4 (ng/dL)
1	1.00	2.56 (0.80–6.26)	7.16 (2.58–5.30)	2.40 (0.81–1.74)
2	1.25	2.13 (0.80–6.26)	12.60 (2.77–4.95)	2.40 (0.81–1.74)
3	1.42	10.71 (0.80–6.26)	11.74 (2.58–5.30)	3.27 (0.81–1.74)
4	1.83	2.25 (0.80–6.26)	7.01 (2.77–4.95)	2.94 (0.81–1.74)
5	2.58	2.72 (0.80–6.26)	8.94 (2.77–4.95)	2.54 (0.81–1.74)
6	3.91	0.91 (0.80–6.26)	5.53 (2.58–5.30)	3.35 (0.81–1.74)
7	7.42	1.68 (0.80–5.40)	10.58 (2.64–4.88)	2.68 (0.82–1.62)
8	8.83	5.20 (0.80–5.40)	10.44 (2.64–4.88)	3.87 (0.82–1.62)
9	9.00	3.86 (0.80–5.40)	8.26 (2.74–4.93)	1.59 (0.82–1.62)
10	9.17	3.92 (0.80–5.40)	8.71 (2.74–4.93)	3.45 (0.82–1.62)
11	9.50	3.34 (0.80–5.40)	23.10 (2.64–4.88)	3.07 (0.82–1.62)
12	10.75	3.50 (0.80–5.40)	5.77 (2.74–4.93)	1.73 (0.82–1.62)
13	10.94	4.69 (0.80–5.40)	16.42 (2.74–4.93)	7.46 (0.82–1.62)
14	11.00	2.24 (0.70–4.61)	9.10 (2.29–4.75)	4.93 (0.81–1.66)
15	12.50	2.15 (0.70–4.61)	10.80 (3.01–4.69)	2.09 (0.81–1.66)

Thyroid function test results were presented with the pediatric age- and sex-specific reference intervals published by Kapelari et al. (10).

### 3.5 Genetic analysis

Genetic analysis identified 12 distinct heterozygous THRB variants in 15 patients (**Table 4**). The variants were confined to exons 10 (n = 8) and 11 (n = 4), encoding the ligand-binding domain of thyroid hormone receptor β. Three variants—c.947G>A (p.Arg316His), c.941C>T (p.Ser314Phe), and c.1286G>A (p.Arg429Gln)—were each identified in two patients, whereas the remaining nine variants occurred in one patient each.

**Table 4:** THRB variants identified in peadiatric cohort (ordered by age at diagnosis).

Patient	cDNA change (HGVS)	Protein change (HGVS)	Zygosity	Exon	Variant significance	Familial history
1.	c.947G>A	p.Arg316His	heterozygous	exon 10	pathogenic	inherited from biochemically affected father
2.	c.1357C>A	p.Pro453Thr	heterozygous	exon 11	pathogenic	mother with biochemical pattern consistent with RTHβ
3.	c.941C>T	p.Ser314Phe	heterozygous	exon 10	pathogenic	familial variant, the same from in brother, parent not tested
4.	c.1286G>A	p.Arg429Gln	heterozygous	exon 11	pathogenic/likely pathogenic	parent not tested, biochemically affected father
5.	c.938T>C	p.Met313Thr	heterozygous	exon 10	pathogenic	N/A
6.	c.947G>A	p.Arg316His	heterozygous	exon 10	pathogenic	N/A
7.	c.990G>C	p.Leu330Phe	heterozygous	exon 10	uncertain significance	inherited from father and grandmother
8.	c.941C>T	p.Ser314Phe	heterozygous	exon 10	pathogenic	familial variant, the same from in brother, parent not tested
9.	c.1286G>A	p.Arg429Gln	heterozygous	exon 11	pathogenic/likely pathogenic	N/A
10.	c.949G>A	p.Ala317Thr	heterozygous	exon 10	pathogenic	parent not tested, biochemically affected mother
11.	c.928A>G	p.Met310Val	heterozygous	exon 10	pathogenic/uncertain significance	N/A
12.	c.1378G>A	p.Glu460Lys	heterozygous	exon 11	pathogenic/likely pathogenic	parent not tested, biochemically affected mother
13.	c.1034G>T	p.Gly345Val	heterozygous	exon 10	pathogenic	N/A
14.	c.1031G>A	p.Gly344Glu	heterozygous	exon 10	pathogenic	N/A
15.	c.1371A>T	p.Glu457Asp	heterozygous	exon 11	uncertain significance	N/A

N/A, Not Applicable/Not Assessed.

### 3.6 Thyroid ultrasound

Thyroid ultrasound findings varied across the cohort (**Table 5**). Normal thyroid morphology was observed in four patients (27%). Enlarged thyroid gland was present in seven patients (47%), heterogeneous and/or hypoechogenic echotexture was present in five patients (33%), while increased vascularity in three patients (20%). Cystic lesions of varying size were detected in seven patients (47%) whereas lesions with solid component in 3 patients (20%).

**Table 5:** Thyroid ultrasound findings in patients with RTHβ.

Thyroid ultrasound finding*	Number (%)
Normal thyroid ultrasound	4 (27%)
Enlarged thyroid gland/goiter	7 (47%)
Heterogeneous and/or hypoechogenic echotexture	5 (33%)
Increased vascularity	3 (20%)
Cystic changes (any size)	7 (47%)
Solid lesions	3 (20%)

*Multiple findings per patient were possible.

### 3.7 Management

Management strategies varied across the cohort. Eight patients (53%) did not receive any pharmacological treatment and were managed conservatively. Beta-blockers were administered in six patients (40%) for symptomatic control of tachycardia. One patient with multiple focal lesions had thyroidectomy and then levothyroxine was started (7%). Five patients (33%) were temporarily treated with methimazole. In three patients (20%), hyperthyroidism was initially diagnosed prior to confirmation of RTHβ. This resulted in the initiation of antithyroid treatment before the correct diagnosis was established. The use of methimazole initially led to improvement in hyperthyroid symptoms; however, it was associated with a progressive increase in TSH concentration and in one case enlargement of the goiter. In one patient with a severe congenital heart defect requiring complex cardiac surgery, methimazole was administered temporarily in the perioperative period, as described in detail in a separate report (11).

Two patients (13%) received bromocriptine. In the first case, bromocriptine was administered at a dose of 1.25 mg. Clinical improvement was observed, including improved psychological well-being, reduced emotional lability, and fewer episodes of tachycardia and normalization of HR. However, no changes in total T4, free T3, or TSH concentrations were observed in laboratory tests. The drug was discontinued after approximately 6 months of treatment due to abdominal pain. Because of hyperactivity, impaired concentration/attention deficits, nervousness, and sleep disturbances, treatment was later reinitiated at a dose of 1.25 mg. A follow-up visit is currently awaited to evaluate treatment efficacy and reassess biochemical parameters.

In the second case, bromocriptine was administered at a dose of 5 mg once daily. Follow-up laboratory tests revealed a decrease in free T4 concentration from 3.28 ng/dL before treatment to 2.09 ng/dL after treatment, T3 from 9.9 pg/mL to 6.83 pg/mL, and TSH from 6.8 mIU/L to 0.68 mIU/L. During treatment, improvement in general well-being, reduced emotional lability, and absence of tachycardic episodes were observed. The treatment was well tolerated, with no reported side effects. After two years of therapy, the dose was reduced to 2.5 mg. Treatment has been continued for 12 years, and the therapeutic effect persists to date.

One patient (7%) was treated with 3,5,3-Triiodothyroacetic acid (TRIAC). A 3-year-8-month-old girl received TRIAC for approximately 5 weeks. Treatment was initiated at 0.35 mg twice daily and subsequently increased to 0.7 mg twice daily. Therapy was generally well tolerated, without deterioration in the patient’s clinical condition. Laboratory follow-up demonstrated a trend toward reduction in TSH, total T4, and free T4 levels. Elevated fT3 concentrations were considered likely related to blood sampling shortly after TRIAC administration. Treatment with TRIAC was discontinued following the development of tachycardia up to 140 bpm during a concurrent upper respiratory tract infection, and symptomatic cardiovascular treatment was intensified.

### 3.8 Follow-up and clinical course

Follow-up data were available for 14 of 15 patients. The median duration of available clinical follow-up was 4.8 years (range, 0.5–13.0 years). Symptoms improved in 7 patients (50%), remained stable in 4 (29%), worsened in 2 (14%), and followed a fluctuating course in 1 (7%). Both improvement and worsening predominantly involved tachycardia and neurobehavioral symptoms, particularly hyperactivity and concentration difficulties. At the last available assessment, 4 patients, representing 27% of the entire cohort, had completed linear growth. Their attained heights ranged from 152.9 to 181.6 cm, corresponding to the 4th–96th percentiles.

## 4 Discussion

This multicenter case series of 15 genetically confirmed pediatric RTHβ patients represents the largest pediatric cohort reported from Poland and Central Europe. While RTHβ is estimated to affect approximately 1 in 19.000-40.000 live births (2), data on clinical presentation in exclusively pediatric populations remain scarce (5, 12). All patients presented with elevated free thyroid hormone concentrations and non-suppressed TSH levels, consistent with the classical biochemical profile (1, 13). TSH-based neonatal screening for congenital hypothyroidism does not reliably detect RTHβ. In the Polish neonatal screening protocol, a dried blood spot TSH concentration of ≥12 mIU/L prompts repeat testing, whereas FT4 and FT3 are not measured in the screening test.Neurodevelopmental disturbances were observed in 60% of our cohort, including psychomotor hyperactivity in five patients (33%), one of whom had a documented ADHD diagnosis; (7%), psychomotor delay and autism spectrum disorder (ASD) in 27% of patients. Thyroid hormones play a key role in brain development, and in RTHβ the coexistence of elevated circulating hormone levels with tissue-specific receptor expression (predominant TRα signaling in the brain) may contribute to an imbalance of thyroid hormone action within the central nervous system, potentially explaining the high frequency of behavioral phenotypes (14). Neuroimaging studies suggest possible structural brain differences in RTHβ, although their clinical relevance remains uncertain (15).

Neurodevelopmental abnormalities have been reported in a substantial proportion of individuals with thyroid hormone resistance. Earlier cohorts described hyperactivity and learning difficulties in approximately two-thirds of affected patients, with the remaining cases showing varying degrees of intellectual impairment (16). Chiesa et al. observed learning disabilities in 60% of their six-patient pediatric cohort, with later diagnoses of attention deficit disorder with or without hyperactivity (5). In contrast, Santos Mata et al. documented behavioral abnormalities in only 13.6% of patients (17). Notably, ASD was documented in four patients (27%).To our knowledge, this observation has not been systematically quantified in previous RTHβ cohorts and may warrant further investigation. Our findings support neurodevelopmental assessment during follow-up of children with RTHβ. They also support considering RTHβ in children presenting with neurodevelopmental or behavioural abnormalities when other clinical features raise suspicion of thyroid dysfunction. In this setting, thyroid function assessment should include TSH, FT4, and FT3, because TSH alone may be within the reference range despite elevated thyroid hormone concentrations.

A striking finding in our cohort was the predominance of a lean phenotype, with 5 of 15 (33%) patients having a BMI below the 3rd percentile for their age and sex, and a further 3 children having a BMI between the 3rd and 10th percentile, giving a total of 53% of patients with a BMI below the 10th percentile. On the other hand, while growth disturbances have been described in RTHβ, no patient in our cohort met the criteria for short stature at diagnosis (18). Only four patients had completed linear growth, and none had a final height below the 3rd percentile. However, this very small subgroup precludes firm conclusions regarding adult height in RTHβ. This pattern underscores the clinical heterogeneity of RTHβ and suggests that alterations in peripheral thyroid hormone action and energy metabolism may influence body composition in affected individuals. Since the TRα receptor (unaffected in RTHβ) is highly expressed in heart, adipose tissue and skeletal muscle, the high circulating thyroid hormones may produce tissue-specific thyrotoxic effects, contributing to hypermetabolism and low BMI despite resistance at the hypothalamic–pituitary level (2).

### 4.1 Genetic insight

All identified variants were located within exons 10 and 11, which encode the ligand-binding domain of THRB, consistent with the known concentration of pathogenic variants in this functionally critical region (19, 20). Variants clustered within two recognized mutational regions of the THRB ligand-binding domain, corresponding to amino acids 310–353 and 429–460. Most common substitutions were observed around codons 313–317. The p.Glu457Asp variant is currently classified as a variant of uncertain significance (ClinVar VCV001809577.1), while p.Leu330Phe has not, to our knowledge, been previously reported in ClinVar, but together with clinical and biochemical symptoms both variants were assessed as causing the disease. Further experimental studies assessing receptor function would help clarify their pathogenic relevance. These considerations imply that patients with resistance to RTHβ often have new, previously undescribed familial variants. Furthermore, it is very important to conduct a bioinformatic assessment of the variants in consultation between the clinician and the geneticist, along with determining the variant’s association with the patient’s clinical data, to interpret the variant correctly.

No apparent genotype–phenotype correlation was observed in our cohort. Patients carrying the same THRB variant showed heterogeneous clinical manifestations (20). A positive family history was documented in eight patients; parental transmission of the THRB variant was genetically confirmed in two patients, while in four additional cases an untested parent showed a biochemical pattern consistent with RTHβ. The occurrence of clinically prominent manifestations in pediatric index cases alongside mild or absent clinical symptoms in biochemically affected relatives is consistent with the variable expressivity of RTHβ (2). Marked phenotypic differences may occur even among carriers of the same THRB variant within a single family. These findings support biochemical screening followed by targeted genetic testing of first-degree relatives, as recommended by the ETA. Distinct THRB variants have been associated with inherited macular dystrophy, but none was identified in our cohort (21). Other clinical findings in our patients cannot be attributed to THRB without further evaluation.

### 4.2 Diagnostic challenges and misclassification

Importantly, 20% of patients in our cohort were initially diagnosed with hyperthyroidism and received methimazole prior to genetic confirmation of RTHβ. As early as 2006, O Olateju et al. reported that failure to differentiate RTHβ from primary thyrotoxicosis had resulted in inappropriate treatment of nearly one-third of patients (22). Antithyroid therapy may further increase TSH secretion and exacerbate goiter development in RTHβ which was also observed in our cohort (23). According to the 2024 European Thyroid Association (ETA) guidelines, antithyroid treatment or thyroid ablation is generally not recommended in patients with RTHβ unless significant comorbidities are present (24).

Despite growing awareness of this condition, our data demonstrate that this diagnostic pitfall remains a relevant clinical problem (22). The key diagnostic clue differentiating RTHβ from primary hyperthyroidism is the non-suppressed TSH in the presence of elevated free thyroid hormones, combined with negative TSH-receptor antibodies. Differential diagnosis should also include TSH-secreting pituitary adenoma, accidental ingestion or intentional or accidental overdose of l-thyroxine and other causes of discordant thyroid function tests, such as assay interference (13). The 2024 ETA guidelines now provide a structured algorithm for the differential diagnosis of discordant thyroid function tests, which should be applied in all such cases (24). Improved recognition of RTHβ may facilitate timely diagnosis, prevent unnecessary treatment, and support individualized management strategies.

### 4.3 Therapeutic considerations

Management of RTHβ is largely symptom-driven and individualized. Importantly, therapeutic decisions in RTHβ should be guided primarily by the patient’s clinical status rather than by attempts to normalize thyroid hormone concentrations. As previously emphasized in the literature, asymptomatic patients generally do not require pharmacological treatment (2). In our cohort, most of the patients (53%) were managed conservatively without therapy, while beta-blockers were used in 40% for tachycardia control - consistent with current recommendations for symptomatic management in RTHβ (24).

In selected cases, additional therapeutic strategies were implemented. One patient was treated with TRIAC, a natural T3 analog. TRIAC may suppress TSH secretion and consequently lower serum FT4 and FT3 concentrations owing to its preferential affinity for TRβ over TRα (25). However, responsiveness to TRIAC appears limited to THRβ mutations located in the carboxy-terminal part of the T3-binding domain (26). Current literature describing the effects of TRIAC remains scarce, including data on possible adverse effects associated with high TRIAC doses in tissues that predominantly express THRα. Nevertheless, existing evidence supports its potential efficacy (27). Carbone et al. reported that TRIAC treatment effectively controlled thyrotoxicosis for approximately five years in a patient with THRβ-related resistance to thyroid hormone, although the therapeutic effect was lost after the development of autoimmune hyperthyroidism (28). Improvement in attention-deficit/hyperactivity disorder (ADHD) symptoms during TRIAC therapy has been reported in children with RTHβ caused by THRB mutations affecting the ligand-binding domain (29, 30). The 2024 ETA guidelines recommend considering TRIAC in patients with predominant symptoms of thyrotoxicosis as well as in children with ADHD (24). Given the high prevalence of tachycardia and neurodevelopmental disturbances in our cohort, TRIAC may be considered in selected symptomatic pediatric patients with RTHβ.

Bromocriptine treatment was used in two patients (31). Bromocriptine, a dopamine receptor agonist, may be considered in RTHβ to help suppress elevated TSH levels through its action on D2 receptors on the pituitary gland. Consistent with its dopaminergic effects, bromocriptine reduces the amplitude of TSH pulses while preserving pulse frequency (32). By reducing pituitary TSH secretion, bromocriptine may contribute to goiter reduction (33). In the case reported by Budzyńska et al., bromocriptine was associated with clinical improvement including better subjective well-being, reduced anxiety, normalization of heart rate, and satisfactory biochemical TSH suppression (31). Combined therapy with bromocriptine and FT4 has been used effectively to lower TSH levels after thyroidectomy in patients with RTHβ (34). Suppressing TSH below the upper limit of the normal reference range in patients with RTHβ is difficult even with very high doses of FT4. Accordingly, combined levothyroxine (LT4) and bromocriptine therapy appears promising, particularly in patients experiencing adverse effects from high-dose FT4 (35).

Neither of our patient treated with bromocriptine required levothyroxine treatment Both experienced clinical improvements, although their biochemical responses differed markedly: the first showed no clear change in FT4, FT3, or TSH, whereas the second showed substantial reductions in all three parameters. It is supported by the available literature that in some patients treated with bromocriptine, clinical improvement has been reported despite the absence of a clear biochemical response. Gu et al. reported three children with RTH, all of whom were treated with bromocriptine (32). All had elevated FT4/FT3 with normal or elevated TSH and clinical features including goiter, irritability, aggressiveness and hyperhidrosis. The authors reported obvious clinical improvement in all three children without apparent adverse effects. However, authors did not provide any evidence on significant biochemical changes in FT4, FT3 and TSH levels. Huang et al. reported a woman with genetically confirmed RTHβ due to the THRB p.Glu460Lys variant whose FT3 and FT4 concentrations did not change significantly after more than 11 months of bromocriptine treatment (36). Recent consensus statements on the management of RTHβ describe bromocriptine as having inconsistent efficacy and note that it is rarely used (37).

Evidence supporting bromocriptine use in RTHβ is limited—particularly in children—and further studies are needed to determine its therapeutic value.

Overall, our findings underscore the heterogeneity of therapeutic approaches in RTHβ and highlight the need for individualized, symptom-oriented management strategies.

### 4.4 Longitudinal clinical course

The longitudinal course in our cohort was heterogeneous, with improvement observed in half of the patients with available follow-up data, while symptoms remained stable, worsened, or fluctuated in others. Given the heterogeneous management and retrospective design, our data do not allow us to determine whether symptoms follow a consistent age-related course. Although published longitudinal data remain limited, the absence of a uniform trajectory is consistent with the variable clinical presentation and outcomes previously described in RTHβ (5, 24).

### 4.5 Study limitations

This study has several limitations, including its retrospective design, small sample size, and heterogeneity in clinical documentation, follow-up duration, and patient management. Nevertheless, given the rarity of RTHβ, this multicenter study provides valuable real-world data from nine pediatric endocrinology centers and represents the largest pediatric RTHβ cohort reported from Poland. The detailed characterization of clinical manifestations, genetic variants, treatment approaches, and longitudinal outcomes broadens the available evidence on the phenotypic and clinical heterogeneity of RTHβ in children.

## 5 Conclusions

This multicenter study of 15 genetically confirmed pediatric patients with RTHβ from Poland expands knowledge of the phenotypic spectrum of this condition in children. Our findings highlight clinically important observations: a high prevalence of neurodevelopmental disturbances including Psychomotor hyperactivity, psychomotor delay and autism spectrum disorder, a predominance of a lean phenotype, a heterogeneous clinical trajectory and variable expression within families. Diagnostic uncertainty remains a relevant clinical issue, as evidenced by the proportion of patients initially misdiagnosed with hyperthyroidism. These findings underscore the importance of considering RTHβ in the differential diagnosis of children presenting with elevated thyroid hormones and non-suppressed TSH, particularly when accompanied by neurobehavioral symptoms. Systematic neurodevelopmental screening, cascade family testing, and adherence to the diagnostic algorithm proposed in the 2024 ETA guidelines may facilitate earlier recognition and prevent unnecessary antithyroid treatment. The potential role of TRIAC in symptomatic pediatric RTHβ warrants further investigation in prospective studies.

## Data Availability

The original contributions presented in the study are included in the article/supplementary material. Further inquiries can be directed to the corresponding author.
